# Neuropilin-1 is over-expressed in claudin-low breast cancer and promotes tumor progression through acquisition of stem cell characteristics and RAS/MAPK pathway activation

**DOI:** 10.1186/s13058-022-01501-7

**Published:** 2022-01-25

**Authors:** Yu Hin Tang, Anja Rockstroh, Kamil A. Sokolowski, Layla-Rose Lynam, Melanie Lehman, Erik W. Thompson, Philip A. Gregory, Colleen C. Nelson, Marianna Volpert, Brett G. Hollier

**Affiliations:** 1 Australian Prostate Cancer Research Centre - Queensland, Institute of Health and Biomedical Innovation, School of Biomedical Sciences, Faculty of Health, Queensland University of Technology, Princess Alexandra Hospital, Translational Research Institute, 37 Kent Street, Woolloongabba, Brisbane, QLD 4102 Australia; 2 Preclinical Imaging Facility, Translational Research Institute, Brisbane, QLD Australia; 3 School of Biomedical Sciences, Faculty of Health and Translational Research Institute, Queensland University of Technology, Brisbane, Australia; 4 Centre for Cancer Biology, SA Pathology and University of South Australia, Adelaide, South Australia Australia; 5 Faculty of Health and Medical Sciences, Adelaide Medical School, University of Adelaide, Adelaide, South Australia Australia

**Keywords:** Neuropilin, Breast cancer, Claudin-low, Triple-negative breast cancer, Cancer stem cells

## Abstract

**Background:**

Triple-negative breast cancers (TNBC) have a relatively poor prognosis and responses to targeted therapies. Between 25 and 39% of TNBCs are claudin-low, a poorly differentiated subtype enriched for mesenchymal, stem cell and mitogen-activated signaling pathways. We investigated the role of the cell-surface co-receptor NRP1 in the biology of claudin-low TNBC.

**Methods:**

The clinical prognostic value of NRP1 was determined by Kaplan–Meier analysis. GSVA analysis of METABRIC and Oslo2 transcriptomics datasets was used to correlate NRP1 expression with claudin-low gene signature scores. *NRP1* siRNA knockdown was performed in MDA-MB-231, BT-549, SUM159 and Hs578T claudin-low cells and proliferation and viability measured by live cell imaging and DNA quantification. In SUM159 orthotopic xenograft models using NSG mice, NRP1 was suppressed by shRNA knockdown or systemic treatment with the NRP1-targeted monoclonal antibody Vesencumab. NRP1-mediated signaling pathways were interrogated by protein array and Western blotting.

**Results:**

High NRP1 expression was associated with shorter relapse- and metastasis-free survival specifically in ER-negative BrCa cohorts. NRP1 was over-expressed specifically in claudin-low clinical samples and cell lines, and NRP1 knockdown reduced proliferation of claudin-low cells and prolonged survival in a claudin-low orthotopic xenograft model. NRP1 inhibition suppressed expression of the mesenchymal and stem cell markers ZEB1 and ITGA6, respectively, compromised spheroid-initiating capacity and exerted potent anti-tumor effects on claudin-low orthotopic xenografts (12.8-fold reduction in endpoint tumor volume). NRP1 was required to maintain maximal RAS/MAPK signaling via EGFR and PDGFR, a hallmark of claudin-low tumors.

**Conclusions:**

These data implicate NRP1 in the aggressive phenotype of claudin-low breast cancer and offer a novel targeted therapeutic approach to this poor prognosis subtype.

**Supplementary Information:**

The online version contains supplementary material available at 10.1186/s13058-022-01501-7.

## Background

Breast cancer (BrCa) is a heterogeneous disease, with genomic profiling studies having identified five major human breast cancer intrinsic subtypes according to PAM50 classification (luminal A, luminal B, HER2-positive, basal-like and normal-like), which differ in their molecular profiles, incidence and prognosis [1]. The most recently classified subtype, claudin-low, was identified in 2007 and is characterized by low expression of cell adhesion proteins such as claudins-3, -4, -7, occludin and E-cadherin, and activation of epithelial-mesenchymal transition (EMT) and mammary stem cell pathways [2, 3]. Claudin-low tumors have the least amount of epithelial differentiation across the breast cancer subtypes and are the subtype that most closely resembles mammary epithelial stem cells [2–4]. Previous studies, including our own [5–7], have revealed claudin-low cell lines and tumors to be enriched in cancer stem cell (CSC) features, overexpressing CSC markers including CD44 and ALDH1, and having low expression of CD24 [3, 8, 9]. Recent analysis suggests that rather than representing a distinct intrinsic subtype, in most cases the claudin-low signature is an acquired secondary phenotype that overlaps with established PAM50 subtypes, typically the basal-like and normal-like groups [10]. Indeed, claudin-low tumors appear to comprise three major subgroups (claudin-low 1, 2 and 3; CL1-3) arising from distinct cells of origin; malignant transformation of normal mammary stem cells (CL1), and EMT-mediated transformation of luminal or basal-like tumors (CL2 and CL3, respectively) [11]. Aside from mesenchymal stem cell properties, claudin-low tumors also exhibit dysregulation of p53 and RAS-MAPK pathways across all cell-of-origin subsets [11]. Claudin-low tumors as a group have a poor response to standard chemotherapy and shorter relapse-free survival and overall survival, although the prognosis of patients with claudin-low tumors reflects the prognosis associated with their underlying intrinsic subtype [3, 10].

The claudin-low subtype is most enriched for basal-like tumors (51.7%), and 25–39% of triple-negative tumors are claudin-low [3, 10]. While hormone therapy and HER2-targeting drugs have improved prognosis of patients with estrogen/progesterone receptor-(ER/PR) and HER2-positive BrCa, respectively, triple-negative breast cancers (TNBCs), including the majority of basal-like tumors, lack all three of these receptors and are poorly responsive to available targeted therapies. Chemotherapy is the mainstay treatment for TNBC, but resistance develops quickly, and apart from of a minor subset of tumors with BRCA mutations that can be treated with PARP inhibitors such as Olaparib (LYNPARZA), there is no targeted therapy for TNBC. These tumors are generally more aggressive than ER/PR- and HER2-positive tumors, while also having a higher incidence in younger women [12]. Despite advances in chemotherapy regimens, TNBC patients still have a relatively poor prognosis with higher recurrence and metastasis rates, and lower survival probability, than other subtypes [13]. Therefore, development of new therapeutic targets for TNBC represents a major unmet clinical need.

Neuropilin-1 (NRP1) is a pleiotropic transmembrane co-receptor protein critical in embryonic development of neurological and vascular systems [14]. It has a short cytosolic segment whose function in intracellular signaling remains unclear, while its extracellular domains mediate interactions with multiple growth factors to promote activation of their cognate receptor tyrosine kinases [15]. Recent evidence suggests that NRP1 activates a broader spectrum of growth factor pathways than formerly thought, including EGF, VEGF, PI3K, HGF, PDGF, FGF and TGF-β1 [16–18]. These interactions have implicated NRP1 in cancer progression across multiple tumor types [15], where high NRP1 expression is associated with poor outcome in lung [19], glioblastoma [20], prostate [21] and breast cancers [22].

We investigated the prognostic value, expression and function of NRP1 across BrCa subtypes. We report that high NRP1 expression predicts shortened time to disease relapse and metastasis in ER-negative patient cohorts and is associated specifically with the claudin-low subtype. NRP1 knockdown in claudin-low cell lines led to significant reduction in cell proliferation and growth of orthotopic claudin-low xenografts. NRP1 over-expression associated with the most de-differentiated claudin-low tumors and was required to maintain high expression of ZEB1 and the mammary stem cell marker ITGA6. Targeted inhibition with an NRP1 monoclonal antibody reduced spheroid-forming capacity and potently suppressed tumor growth in an orthotopic claudin-low TNBC xenograft model. Finally, we show that NRP1 acts as a central hub for the aberrant activation of the RAS-MAPK pathway via EGFR and PDGFR activation to drive aggressive tumor progression, a hallmark of all claudin-low subgroups. These data identify NRP1 as a key driver of the claudin-low phenotype and support further testing of NRP1 inhibitors for improved control of claudin-low tumor progression.

## Materials and methods

### Survival analysis

*NRP1* expression correlation with relapse-free survival (RFS) and distant metastasis-free survival (DMFS) was analyzed using Kaplan–Meier Plotter software (https://kmplot.com/analysis/index.php?p=service&cancer=breast) with the following non-default settings selected; Affymetrix ID / Gene symbol; 212298_at, Split patients by; upper quartile, Survival; RFS (4934) or DMFS (2767), Probe set options; JetSet best probe set, ER status—array (*n* = 7535); all, ER positive (5526) or ER negative (2009) [23, 24]. The expression of NRP1 in the Cancer Genome Atlas Breast Invasive Carcinoma (TCGA-BRCA) data set and relevant clinical parameters was downloaded from the University of California Santa Cruz (UCSC) Xena data portal (https://xena.ucsc.edu). For Kaplan–Meier analysis, patients were ranked by NRP1 expression from lowest-to-highest then divided into quartile groups (Q1-Q4), with Q4 being the highest expressing patients, and median months overall survival computed per quartile group of patients.

### In silico analysis

Molecular Taxonomy of Breast Cancer International Consortium (METABRIC) and The Cancer Genome Atlas (TCGA) datasets were accessed through cBioPortal and UCSC XENA [25] [26]. NRP1 expression was correlated to claudin-low up- and down-signature scores with RStudio (version 3.5.1) GSVA package [27] using published claudin-low gene signatures [3, 10]. Core claudin-low (CoreCL) signature was obtained from Fougner et al. [10]. Heatmaps were generated using Morpheus software (Broad Institute).

The Prediction Analysis of Microarrays (PAM) R package (pamr) was used to classify the three claudin-low subtypes using the ‘nearest shrunken centroids’ algorithm as reported by Pommier et al. [11]. The prediction model was trained based on expression of the 137 classifier genes in a subset of 45 claudin-low samples, as provided by Pommier et al. The trained model was applied to the 199 claudin-low samples from the METABRIC dataset to classify them into CL1-3 subtypes for NRP1 expression analysis. The ggplot and ggplot2 R packages were used to generate the graphical presentation for the claudin-low subtype analysis. NRP1 expression in BrCa cell lines was analyzed using transcriptomic data from Neve et al. [28].

### Cell culture

MDA-MB-231, BT-549, MCF-7, T47D, HS578T and SUM159 BrCa cell lines were sourced from the American Type Culture Collection (ATCC; Manassas, VA, USA). MDA-MB-468, BT-20, MDA-MB-361, BT474, SKBR3 and HCC1569 cells were obtained from the ATCC and transferred from Lombardi Cancer Center, USA, by Prof. Erik Thompson (QUT). All cell lines were authenticated by short tandem repeat analysis at the Genomics Research Centre (Queensland University of Technology, Australia). SKBR3, HCC1569 and T47D cells were cultured in RPMI 1630 (Gibco) supplemented with 10% FBS (Gibco). MDA-MB-231, SUM159P, HS578T, BT-549, MDA-MB-468, MDA-MB-361 and BT474 cells were cultured in DMEM (Gibco) with 10% FBS. MCF-7 cells were cultured in DMEM (Gibco) with 10% FBS and 10 ug/mL insulin (Gibco).

### Western blotting

Cells were harvested in lysis buffer (1% Triton-X, 150 mM NaCl, 1 mM EDTA, 50 mM Tris, pH 8) containing protease and phosphatase inhibitors (cOmplete Protease Inhibitor Cocktail and PhosSTOP; Roche) on ice for 30 min. Protein concentration was determined by the Pierce BCA Protein Assay Kit (Bio-Rad). Protein samples were prepared using Bolt LDS Sample Buffer and Reducing Agent (Thermo Fisher). Samples were heated at 70 °C for 10 min and separated on 4–12% sodium dodecyl sulfate polyacrylamide gel electrophoresis (SDS-PAGE) gels (Thermo Fisher). Proteins were transferred onto a nitrocellulose membrane using the Mini Blot Module (Thermo Fisher Scientific). Membranes were blocked in Tris-buffered saline containing 0.05% Tween 20 (TBS-T) and 5% non-fat powdered skim milk for one hour, then incubated in primary antibody overnight as follows; NRP1 (Santa Cruz Biotechnology, C-19/sc-7239, 4 µg/ml), GAPDH (Cell Signaling Technology, D16H11/5174), EGFR (Cell Signaling Technology, D38B1/4267), phospho-EGFR (Cell Signaling Technology, D7A5/3777), γ-tubulin (Sigma Aldrich, T5326, 1 µg/ml), ZEB1 (Sigma Aldrich, HPA027524), p42/44 (Cell Signaling Technology, 137F5/4695), phospho-p42/44 (Cell Signaling Technology, D13.14.4E/4370), ITGA6 (Cell Signaling Technology, 3750), PDGFR (Cell Signaling Technology, D1E1E/3174) or phospho-PDGFR (Cell Signaling Technology, 4547) according to manufacturer’s instructions. After washing with TBS-T, horseradish peroxidase-conjugated secondary antibodies were applied for one hour at room temperature in 5% skim milk and immunodetection performed using Immobilon TM Western Chemiluminescent HRP Substrate (Millipore). Chemiluminescent signal was visualized using the Konica SRX-101A film processor or Chemidoc Gel Imaging System (BioRad). Densitometry was performed using Image Studio Lite software.

### Quantitative PCR

RNA was extracted with the Direct-zol RNA miniprep kit (Zymo Research) before reverse transcription with the SensiFAST cDNA Synthesis kit (Bioline). Quantitative PCR was performed using SYBR Green (Thermo Fisher) using QuantStudio 6 Real-Time PCR System (Thermo Fisher). Gene expression was determined by the comparative Ct method, and normalized to the housekeeping gene RPL32. Expression in NRP1 knockdown samples was normalized to NT control. The following primer sequences (5’-3’) were used: NRP1 (forward; CGAGGGCGAAATCGGAAAAGG, reverse; CTTCGTATCCTGGC*GTGCT*), ZEB1 (forward; CAACTACGGTCAGCCCT, reverse; GCGGTGTAGAATCAGAGTC), ITGA6 (forward; *CCTCTTCGGCTTCTCGCTG*, reverse; *CGTGGGGTCAGCATCGTTA*).

### Flow cytometry

For NRP1 expression analysis, cells at 80% confluence in 75 cm^2^ cell culture flasks were washed with ice-cold PBS and dislodged with Accutase (Gibco). Cells were neutralized with PBS/5% FBS and resuspended to 10^6^ cells/ml. Anti-human APC-conjugated NRP1 antibody (R&D System, FAB3870A) or IgG-APC control (R&D System, IC003A) was added and cells incubated on ice for 45 min. Cells were centrifuged, resuspended in PBS/5% FBS and propidium iodide (100ug/ml) added. Fluorescence compensation and analysis was performed on a BD Accuri C6. Results were analyzed with Kaluza software. The X-axis parameter Median (‘X-Med’; the 50th percentile of a population, representing the value at which half of a measured population is above and the other half below) was used to represent fluorescence intensity. For CD44/CD24 based cell sorting, 20 × 10^6^ cells were grown to 80% confluence, washed twice in PBS and detached with Accutase (Thermo Fisher). After washing and resuspension (10^6^ cells/ml) in PBS/5% FBS, the cell suspension was incubated with anti-human CD44-Alexa Fluor 488 (FAB6127G, R&D Systems)) and CD24-APC (FAB5247A, R&D Systems) for 1 h on ice, then washed three times in PBS/5% FBS. Propidium iodide (3 μl, 100 μg/ml) was added to the cells immediately before loading on a MoFlo Astrios Cell Sorter (Beckman Coulter) to allow for viable cell gating.

### siRNA and shRNA knockdown

BrCa cells were reverse transfected using Lipofectamine RNAiMax reagent (Thermo Fisher Scientific) and 5 nM Silencer Select siRNA (Ambion) according to manufacturer’s instructions. The following NRP1 siRNA sequences were used (sense, 5’-3’): siNRP1 [1]; uaaccacauuucacaagaa, siNRP1 [2]; cagccuugaaugcacuuau. A pre-designed Silencer Select non-targeting (NT) siRNA was used as a negative control (Negative Control siRNA No. 1, #4,390,844, Thermo Fisher Scientific). For western blotting, protein lysate was collected 72 h post-transfection. For constitutive knockdown, an NRP1 shRNA pLKO.1 lentiviral vector with NRP1 anti-sense sequence 5ʹ-AATACTAATGTCATCCACAGC-3ʹ was used, with the control sequence targeting firefly luciferase (shCntrl) obtained from Thermo Fisher. Viral particles were produced as previously described [5].

### Proliferation assays

Cell viability was measured at 0, 1, 2, 4 and 7 days post-siRNA treatment. A total of 5,000 cells were plated per well of a 96-well plate after mixing with transfection solution as described. Viability was assessed using the CyQuant Direct Cell Proliferation Assay (Life Technologies) or Incucyte S3 (Essenbioscience) according to manufacturer’s instructions. For day 0 measurement, cell viability was quantified 6 h after cells were plated.

### Spheroid assays

Spheroid formation assays were carried as described previously [29, 30]. Briefly, 1,200 BrCa cells in single cell suspension were seeded into ultra-low attachment 96-well plates (Corning) in standard growth medium containing 1% methylcellulose, 1 × B-27™ supplement (Thermo Fisher Scientific), EGF (20 ng/mL), bFGF (20 ng/mL) and heparin (4 μg/mL). Vesencumab or IgG was added to wells to a final concentration of 50 µM and replenished daily. Whole well images were captured (4x) every 24 h by the Incucyte® S3 Live-Cell Analysis System. Image resolution was set as 2400 × 2400 pixels with 2.8 pixel to μm ratio. Images were exported and spheroid number analyzed by CellProfiler (https://cellprofiler.org/) based on spheroid diameter ≥ 50 μm or ≥ 18 pixels.

### Experimental animals

All animal studies were carried out with approval from the University of Queensland Animal Ethics Committee (ethics approval number QUT/TRI/026/18). Eight-week-old female NSG (NOD.Cg-*Prkdc*^*scid*^* Il2rg*^*tm1Wjl*^/SzJ) mice were purchased from the Animal Resources Centre (ARC), Western Australia, and acclimatized for one week at the Biological Resources Facility (BRF), Translational Research Institute, before beginning experiments. Mice were maintained on standard irradiated rat and mouse diet (Specialty Feeds) and provided a 12 h light/12 h dark cycle. Twice a week, mice received sunflower seeds as a supplement. Mouse health was monitored at least three times weekly by researchers and daily by BRF staff. All procedures were performed in a sterile laminar flow cabinet.

### Orthotopic claudin-low breast cancer xenograft models

1 × 10^6^ SUM159 cells constitutively expressing luciferase (SUM159^luc^) in 30 µL Matrigel (Corning) were injected into the left inguinal mammary fat pad of 8-week-old female NOD SCID gamma (NSG) mice. To test the effects of shRNA NRP1 knockdown, SUM159^luc^ transfected with shNRP1 or shNT were used for injection (10 mice per group). Mice in the shRNA study were allowed to progress to ethical endpoint (tumor volume 1000 mm^3^). To test the effects of the NRP1 inhibitor Vesencumab, mice injected with SUM159^luc^ cells were randomly allocated to IgG control or Vesencumab treatment groups (10 mg/kg by twice weekly intraperitoneal injection until study endpoint; 12–14 mice per group), for 7 weeks. Bioluminescence imaging of tumors was performed with the IVIS Spectrum In Vivo Imaging System (Perkin Elmer) one-week post-xenografting to confirm presence of live tumor cells. Tumor size was measured by digital caliper twice weekly until endpoint, when bioluminescence imaging of tumors was repeated prior to tissue collection. Vesencumab and IgG control were provided by Genentech Inc.

### Immunohistochemistry

Paraffin sections were dewaxed, rehydrated and then underwent antigen retrieval in 1 mM EDTA buffer (pH 9) in a microwave oven (700 W) for 16 min. Endogenous peroxidase block was performed for 15 min in 3% hydrogen peroxide, followed by 3 washes of 10 min each with phosphate buffered saline (PBS) containing 0.1% Tween-20 (PBS-T). Sections were blocked for 30 min with CAS-block reagent (Zymed Laboratory), followed by 3 PBS-T washes. Slides were incubated with the following primary antibodies diluted in Dako antibody diluent at 4 ^0^C for 16 h as follows; NRP1 (Sigma Aldrich, HPA030278, 4 µg/ml), Ki67 (Agilent, MIB-1/M7240), CD31 (Abcam, ab28364) and phospho-p42/44 (Cell Signaling Technology D13.14.4E/4370), with Ki67, CD31 and phospho-p42/44 antibodies diluted according to manufacturer’s instructions. Slides were washed, incubated with EnVision + Dual Link System HRP (Agilent) for 30 min, washed again and developed in Liquid DAB + Substrate Chromogen System (Agilent). Slides were counterstained with Mayer’s hematoxylin, dehydrated and mounted in DPX. Slides were scanned by the Panoramic Digital Slide Scanner (3DHISTECH) and analyzed with CaseViewer software using QuantCenter (3DHISTECH). The PatternQuant module was used to discriminate epithelial, stromal and necrotic compartments. DensitoQuant (NRP1, CD31) or NuclearQuant (Ki67, phospho p42/44) modules were used for quantification of IHC staining. For NRP1 IHC quantification, the following DensitoQuant settings were used: Detection (Brown Tolerance; 1.2, Blue Tolerance; 0.98), Score (Weak Positive Intensity; 220, Moderate Positive Intensity; 180, Strong Positive Intensity; 150). For phospho p42/44 quantification, the following NuclearQuant settings were used: Nucleus Detection (blur; 15, radius; 3–8, min area; 10), Nucleus Filters (intensity; 60, contrast; 30), Score (0/negative; 255–200, + 1/weak; 200–164, + 2/medium; 164–120, + 3/strong; 120–0).

### Receptor tyrosine kinase (RTK) array and Vesencumab treatment

Cells were seeded into T25 flasks and serum-starved for 16 h when they reached 70% confluence. Cells were pre-incubated with Vesencumab or IgG control (50 μg/mL) for 1 h followed by receptor activation by addition of culture medium containing 20% FBS for 60 min. For NRP1 knockdown study, cells were transfected and seeded as described above. Two days post-transfection, cells were serum starved for 24 h followed by receptor activation by addition of culture medium containing 20% FBS for 0, 10 or 60 min. Protein lysates were then collected and antibody array binding performed according to manufacturer’s instructions (Proteome Profiler Human Phospho-RTK Array Kit, ARY001B, R&D Systems). Antibody membranes were scanned by ChemiDoc (Bio-Rad) and densitometry performed using Image Lab software (Bio-Rad).

### Statistical analyses

Differences between two groups were compared using unpaired Student’s t test. For multiple group comparisons, one-way analysis of variance (ANOVA) followed by Dunnett’s post hoc test was used; for survival analysis, log-rank (Mantel-Cox) test was used to assess the significant differences among treatment and control groups. For in silico analyses, Chi-square test was used to assess the significance between subtypes, and linear regression analysis was used to calculate the significance between NRP1 expression and gene score. Tumor Control Index (TCI) was used to estimate the tumor size for one mouse (shCntrl group; shNRP1 in vivo study) which was culled early for health reasons before to reaching ethical tumor volume endpoint. The Srivastava Lab kindly provided the VBA macro with graphical user interface (SL TCI) that calculates TCI scores, based on tumor rejection, regression and stability scores [31].

## Results

### NRP1 expression is prognostic of relapse and distant metastasis in ER-negative breast tumors

**Fig. 1:**
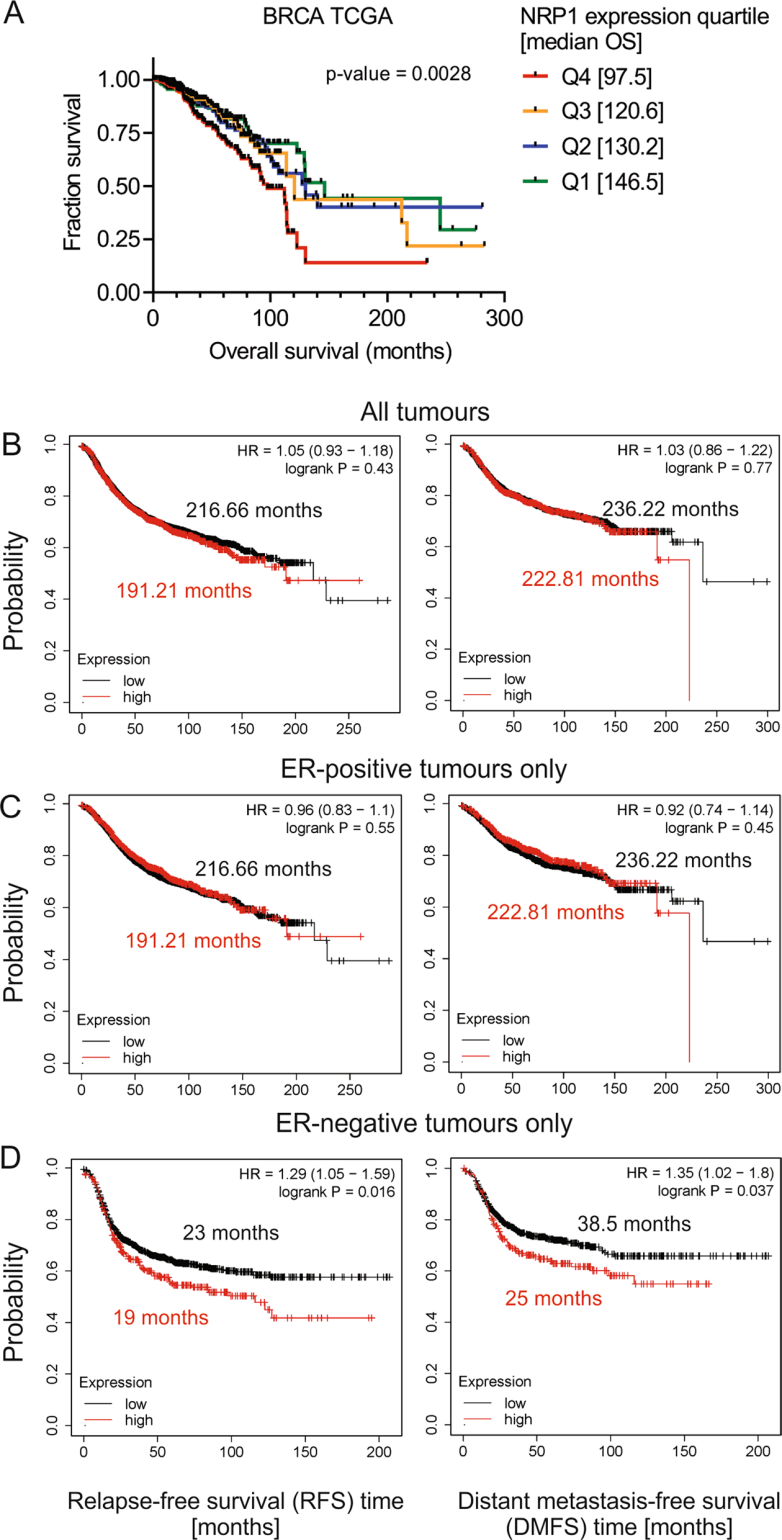
High NRP1 expression predicts shorter time to relapse- and distant metastasis-free survival in ER-negative breast cancer patient cohorts. **a** Association of NRP1 expression quartiles (Q1–Q4) with overall survival in the BrCa TCGA cohort [33]. Data were obtained from UCSC Xena [32]. KM Plotter analysis of relapse-free survival (left panel; RFS) and distant metastasis-free survival (right panel; DMFS) in an **b** unselected patient cohort (RFS; *n* = 4929, DMFS; *n* = 2765; months survival displayed as median survival), **c** ER-positive only (RFS; *n* = 3768, DMFS; *n* = 2016; months survival displayed as median survival) and **d** ER-negative only (RFS; *n* = 1161, DMFS; *n* = 749; months survival displayed as upper quartile survival) tumor subcohorts [23, 24]

To assess whether NRP1 expression has utility as a prognostic marker of BrCa outcome, we utilized the TCGA breast cancer dataset. A significant correlation (logrank test for trend *p* = 0.0028) was observed between high NRP1 expression (Q4) and shorter overall survival time (Fig. 1a) [32, 33].

To delineate BrCa subtypes where NRP1 expression had the greatest prognostic value, the KM Plotter database (http://kmplot.com/analysis/) was used to interrogate a combined dataset comprising 50 independent BrCa patient cohorts [23, 24]. High NRP1 expression (upper quartile) was not significantly associated with shorter relapse-free survival (RFS) (*p* = 0.43; HR 1.05) or distant metastasis-free survival (DMFS) (*p* = 0.77; HR 1.03) across an unselected cohort of patient tumors (Fig. 1b).

When patients were stratified according to positive or negative ER status, NRP1 association with shorter time to RFS was more pronounced in ER-negative patients (Fig. 1d; *p* = 0.016; HR 1.29), while no significant difference was seen in ER-positive patients (Fig. 1c; *p* = 0.55; HR 0.96). For DMFS, high NRP1 levels were most strongly associated with poor outcome in ER-negative patients (Fig. 1d; *p* = 0.037; HR 1.35), whereby ER-negative patients with NRP1 expression in the upper quartile had a relatively short time to distant metastasis (upper quartile survival 25 versus 38.5 months). Hence, elevated levels of NRP1 expression were indicative of poor BrCa patient outcomes, particularly in ER-negative patient cohorts.

### NRP1 over-expression is associated with claudin-low breast cancer

**Fig. 2:**
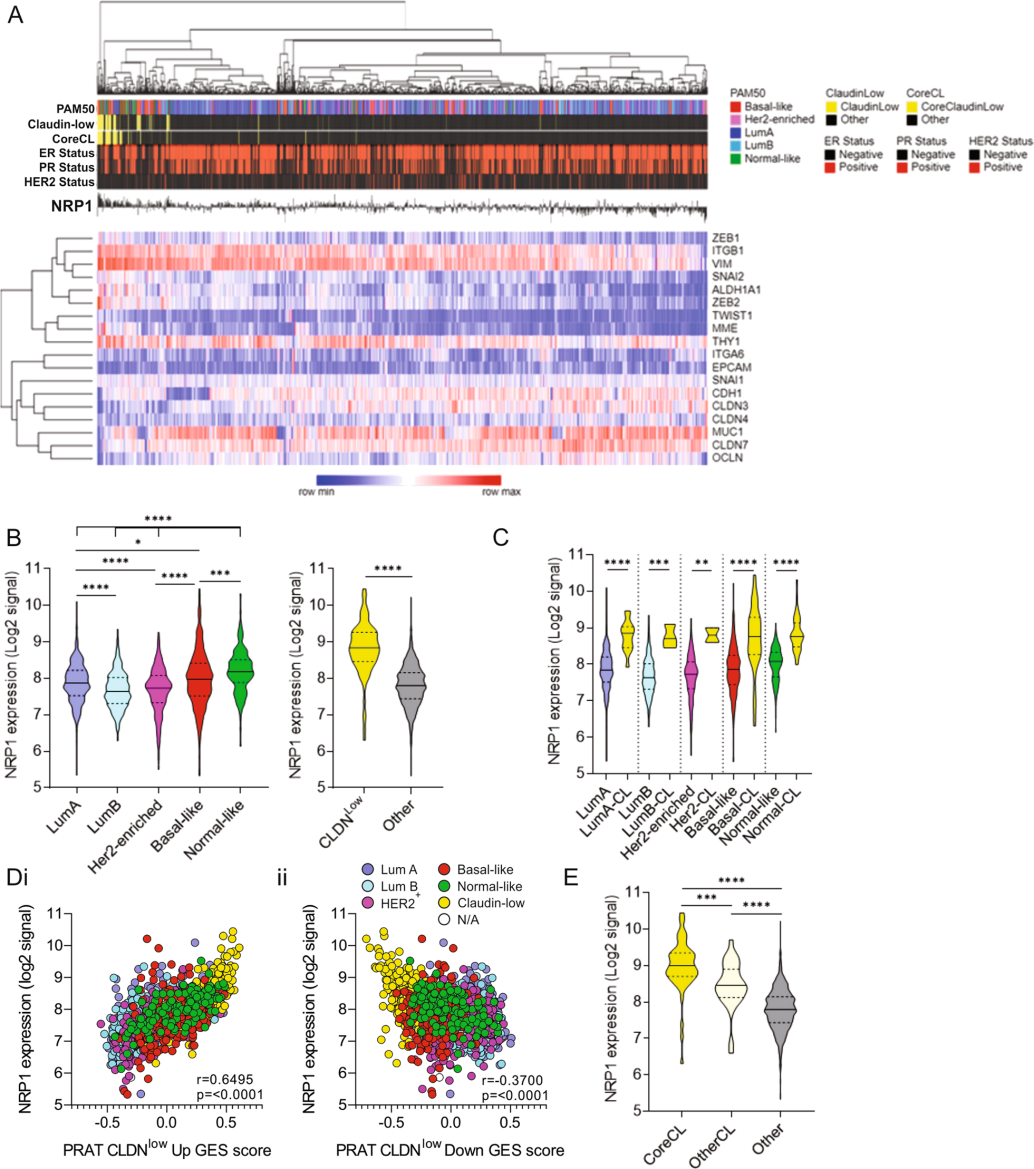
NRP1 is over-expressed in the claudin-low molecular subtype of breast cancer. **a** Heatmap showing NRP1 expression association with PAM50, claudin-low, core claudin-low (CoreCL), ER and HER2 tumor status, as well as core claudin-low signature genes. **b** NRP1 mRNA expression (log2 signal) in intrinsic breast cancer subtypes and claudin-low tumors (CLDN^low^) in the METABRIC patient dataset (*n* = 1904), obtained through cBioPortal [25]. **c** NRP1 mRNA expression across intrinsic subtypes subdivided into claudin-low (CL) and non-claudin-low tumors. Correlation of claudin-low **di** up-gene (CLDN^low^ UP GES) and **dii** down-gene (CLDN^low^ DN GES) GSVA-derived signature scores with NRP1 expression. Claudin-low gene signature scores were obtained via GSVA. Sample subtype is represented according to color scheme used in A-C. **e** NRP1 mRNA expression in METABRIC core claudin-low (CoreCL), non-core claudin-low (OtherCL) and non-claudin-low tumors [10]. Error bars represent SEM, *p ≤ 0.05; **p ≤ 0.01; ***p ≤ 0.001; ****p ≤ 0.0001

To investigate NRP1 expression in ER-negative tumors, we assessed *NRP1* transcript levels across BrCa subtypes in publicly available clinical BrCa datasets. The association of NRP1 with ER-negative status was validated in the METABRIC dataset (Fig. 2a). Interestingly, in silico analysis of the METABRIC dataset [25] revealed that *NRP1* expression was significantly higher in claudin-low than any other BrCa subtype (Fig. 2a, b). NRP1 expression was also elevated in normal-like tumors, possibly reflecting the reported enrichment of the normal-like subtype among claudin-low tumors (Fig. 2b) [3, 10]. *NRP1* expression was significantly elevated in claudin-low tumors within each intrinsic subtype, compared with their non-claudin-low counterparts (Fig. 2a, c). Significant correlations between *NRP1* expression and both claudin-low up- and down-gene signatures scores as reported by Prat et al. [3] were observed, although the association with claudin-low-up gene signature score was more prominent (Fig. 2di).

A recently published condensed claudin-low 19-gene list (‘Core CL’) identifies the core cell–cell adhesion, EMT and stem cell-like pathways that are hallmarks of the claudin-low subtype [3, 10]. *NRP1* remained significantly over-expressed in core claudin-low (CoreCL) tumors compared to non-core claudin-low and non-claudin-low tumors (Fig. 2a, e). The association of *NRP1* expression and the core claudin-low signature was maintained in the Oslo2 cohort [34], suggesting a role of NRP1 in promoting or maintaining mesenchymal and/or stem cell characteristics in claudin-low tumors (Fig. S1).

### NRP1 is over-expressed in claudin-low cell lines and promotes tumor cell proliferation

**Fig. 3:**
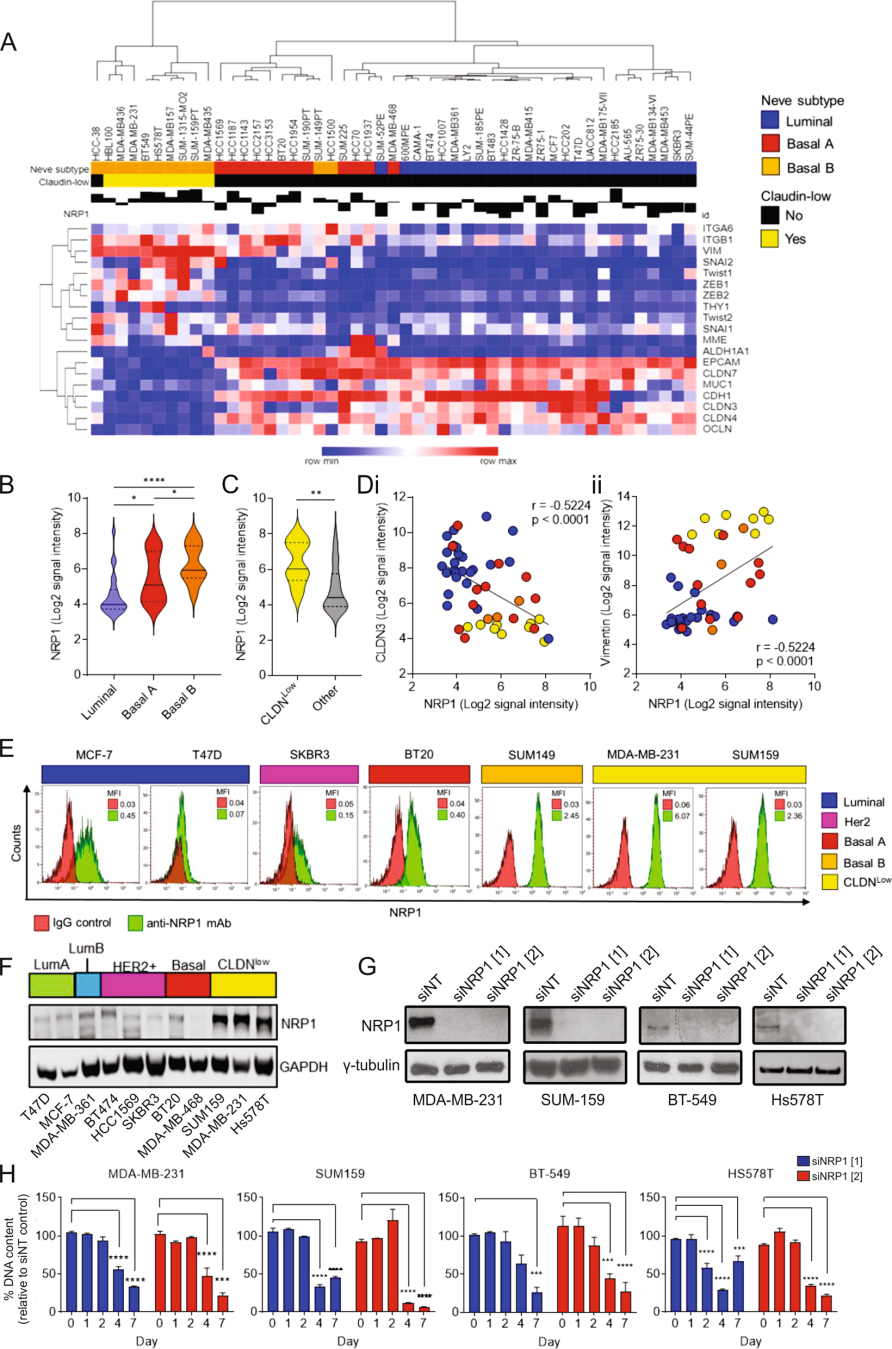
NRP1 is associated with the claudin-low signature in breast cancer cell lines and promotes proliferation. **a** Heatmap showing NRP1 mRNA expression across luminal, basal A, basal B and claudin-low human breast cancer cell lines, as well as association with core claudin-low signature genes [10, 28]. NRP1 mRNA expression in **b** luminal, basal A and basal B breast cancer cell lines, **C** claudin-low versus non-claudin-low cell lines, and association with key claudin-low markers **di** claudin 3 (CLDN3) and **dii** vimentin across breast cancer cell lines. **E** Flow cytometry analysis of NRP1 expression across breast cancer cell lines representing different intrinsic subtypes and the claudin-low (CLDN^low^) subtype. **f** Western blot analysis of NRP1 expression in BrCa cell lines including luminal A (LumA; T47D and MCF-7), luminal B (LumB; MDA-MB-361), HER2^+^ (BT-474, HCC1569 and SKBR3), basal (BT-20 and MDA-MB-468) and claudin-low (SUM159, MDA-MB-231 and HS578T) cells, with GAPDH loading control. **g** Western blot showing NRP1 expression in claudin-low cell lines (MDA-MB-231, BT549, SUM159 and HS578T) at day 3 post-transfection with NRP1 siRNA (siNRP1 [1] or siNRP1 [2]) or non-targeting control siRNA (siNT). **h** Cell viability of claudin-low cell lines (MDA-MB-231, BT549, SUM159 and HS578T) at 0, 1, 2, 4 and 7 days after transfection with NRP1 siRNA (siNRP1 [1] or siNRP1 [2]) relative to siNT as measured by CyQuant™ DNA quantification assay; *n* = 3**.** Error bars represent SEM, *p ≤ 0.05; **p ≤ 0.01; ***p ≤ 0.001, ****p ≤ 0.0001

Next, we screened a panel of cell lines representing a range of BrCa molecular subtypes as per Neve et al. [28] for *NRP1* expression (Fig. 3a). *NRP1* expression was lowest in cell lines from the luminal subtype and highest in basal B cell lines (Fig. 3b). All claudin-low cell lines were of the basal B subtype, and *NRP1* was significantly more highly expressed in claudin-low cell lines than non-claudin-low (Fig. 3c). *NRP1* mRNA expression correlated significantly with core claudin-low markers, including inverse and positive associations with claudin-3 and vimentin, respectively (Fig. 3d). Both flow cytometry (Fig. 3e) and Western blotting (Fig. 3f) confirmed up-regulation of NRP1 protein levels in claudin-low cell lines. NRP1 inhibition using two targeted siRNA sequences (siNRP1 [1] and siNRP1 [2]) and a non-targeting (siNT) control sequence in claudin-low cell lines MDA-MB-231, BT-549, SUM159 and HS578T was effective in suppressing NRP1 expression (Fig. 3g). DNA content quantification showed that NRP1 knockdown caused a marked decrease in cell proliferation from post-transfection day 4 onwards across all claudin-low cell lines tested (Fig. 3h). These results were validated in MDA-MB-231 cells using two additional NRP1-targeting siRNA sequences (siNRP1 [3] and [4]) (Additional file 1: Fig. S2).

### NRP1 expression is associated with in vivo claudin-low xenograft progression and cancer stem cell properties

**Fig. 4:**
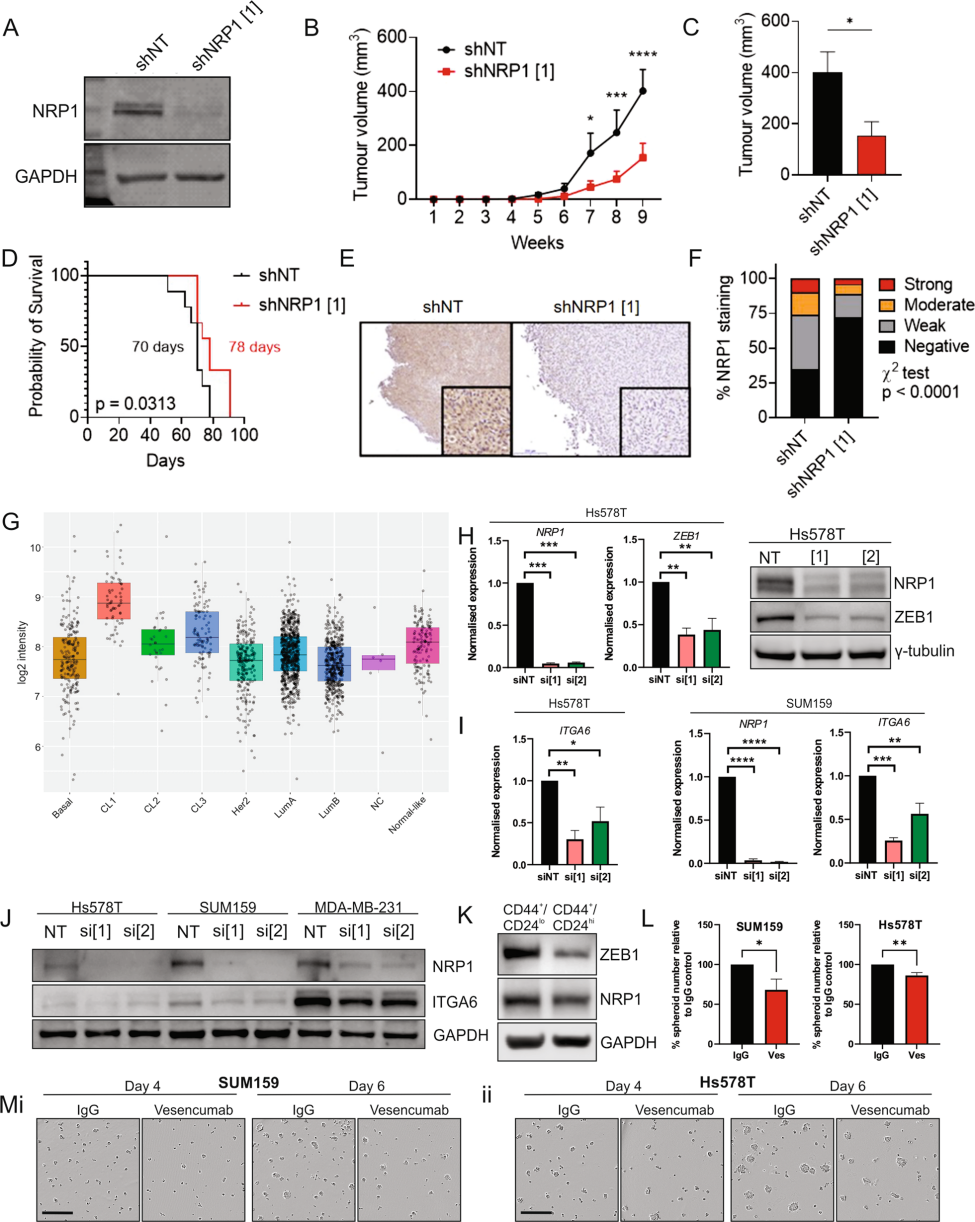
NRP1 expression is associated with in vivo tumor progression, cancer stemness and spheroid-initiating potential. **a** Western blot analysis of NRP1 expression in non-targeting control (shNT) and NRP1 shRNA-silenced (shNRP1 [1]) SUM159 cells inoculated into mice. **b** Post-inoculation tumor volumes, **c** tumor volume at week 8 post-inoculation and **d** Kaplan–Meier analysis of overall survival in SUM159 shNT and NRP1 knockdown groups. **e** Representative images of NRP1 immunohistochemistry in shNT and shNRP1 tumors and **f** quantification of NRP1 IHC staining across shNT and NRP1 knockdown groups. **g** NRP1 expression across CL1, CL2 and CL3 claudin-low subtypes as well PAM50 classifiers in the METABRIC dataset obtained via cBioportal [11, 25]. **h** qPCR (left and center panel; *n* = 3) and Western blot (right panel) analysis of ZEB1 expression in HS578T cells after 72 h NRP1 knockdown versus NT control. **i** qPCR analysis of ITGA6 mRNA expression in HS578T cells (leftmost panel; NRP1 expression shown in **h**) and SUM159 cells (center and right panel) after 72 h NRP1 knockdown versus NT control (*n* = 3). **j** Western blot showing ITGA6 expression in HS578T, SUM159 and MDA-MB-231 cells after 72 h NRP1 knockdown versus NT control. **k** Western blot showing expression of ZEB1 and NRP1 in FACS sorted CD44^+^/CD24^lo^ and CD44^+^/CD24^hi^ populations of SUM159 cells. **l** Number of spheroids (> 50 µM) formed by day 6 following seeding of single cell SUM159 and Hs578T cell cultures containing 1,200 cells in the presence of 50 µg/ml Vesencumab (red lines) or IgG control (black lines). *n* = 5, along with **m** representative images of (**mi**) SUM159 and (**mii**) Hs578T spheroid cultures at days 4 and 6 post-seeding. *p ≤ 0.05; **p ≤ 0.01; ***p ≤ 0.001, ****p ≤ 0.0001, error bars represent SEM

To confirm the growth suppressive effects of NRP1 knockdown on claudin-low cells in vivo, constitutive NRP1 knockdown SUM159 cells were generated by lentiviral transfection with an NRP1-targeted shRNA sequence shNRP1 [1] or a control scrambled shRNA sequence (shNT). One-week post-transfection, 1 × 10^6^ shNRP1 or control shNT SUM159^luc^ cells were orthotopically injected into mammary fat pads of female NSG mice and tumor growth was monitored until ethical endpoint (tumor volume 1000 mm^3^) was reached. NRP1 expression was suppressed in shNRP1 cells at the time of injection, as confirmed by Western blotting in the cells remaining after xenografting (Fig. 4a). Tumor growth was suppressed in mice in the shNRP1 group compared to control, and tumor volumes from post-inoculation week 7 onwards were significantly lower in the NRP1-suppressed group (Fig. 4b–c). Overall survival time was significantly longer in the shNRP1 group (Fig. 4d). NRP1 expression was confirmed to be reduced in the shNRP1 group endpoint tumors compared to shNT control by immunohistochemistry, with NRP1 antibody validation performed in NRP1 knockdown SUM159 cells (Fig. 4e–f, Additional file 1: Fig. S3). These data implicate NRP1 in promoting aggressive claudin-low tumor proliferation and suggest that therapeutic inhibition of NRP1 may suppress the growth of claudin-low tumors.

A gene classifier identifying 3 molecular subtypes of claudin-low BrCa (CL1, CL2 and CL3) has recently been defined [11]. In their genetic and molecular profile, CL1 tumors resemble mammary stem cells and have been posited to arise from their malignant transformation, while CL2 and CL3 tumors more closely resemble luminal and basal tumors, respectively [11]. Using the claudin-low subtype gene classifier to allocate claudin-low molecular subtypes to claudin-low tumors from the METABRIC dataset, we found that *NRP1* expression was significantly higher in CL1 than CL2 and CL3 subtypes, suggesting that NRP1 may maintain stem cell characteristics in mammary cells (Fig. 4g) [11, 25]. Among the claudin-low cell lines tested, Hs578T cells best represent the CL1 subtype [11]. Since ZEB1 over-expression is characteristic of the CL1 subtype, we investigated whether NRP1 could regulate ZEB1 expression in Hs578T cells [11]. Indeed, NRP1 knockdown in Hs578T cells markedly reduced ZEB1 mRNA and protein levels (Fig. 4H). Assessment of additional EMT markers Zeb2, Vimentin, Snail, Slug, Twist, EpCAM and E-Cadherin following NRP1 knockdown with siNRP1 was also performed. While expression of most markers did not change significantly, the most consistent changes observed were in *ZEB2* and *VIM* expression levels across claudin-low cell lines (Additional file 1: Fig. S4). In Hs578T cells, *TWIST1* was also significantly reduced following NRP1 knockdown, but this was not the case in SUM159 and MDA-MB-231 cells (Additional file 1: Fig. S4).

Next, we investigated whether NRP1 knockdown could modulate the expression of mammary stem cell markers. Both qPCR and Western blotting following NRP1 knockdown revealed that NRP1 up-regulates ITGA6/CD49f, a well-established marker of the mammary stem cell population, in claudin-low cells (Fig. 4i–J) [35]. ITGA6 maintains multipotency of mammary stem cells, and selection of mammary cells expressing high ITGA6 enriches for stem cells capable of regenerating mammary tissue in vivo [35–38]. Hence, we speculated that NRP1 may influence stem cell properties such as self-renewal. Selection of a CD44^+^/CD24^low^ subpopulation can reportedly enrich for a subpopulation of cells with self-renewal potential [39]. To determine if NRP1 expression is enriched in the CD44^+^/CD24^low^ population, SUM159 cells were sorted into CD44^+^/CD24^low^ and CD44^+^/CD24^high^ populations. Concomitant with a reduction in ZEB1, NRP1 levels were slightly reduced in CD44^+^/CD24^high^ cells (Fig. 4k). Although MDA-MB-231 cells were found to have very low numbers of CD24^+^ cells (> 1%), qPCR analysis of NRP1 in CD44^+^/CD24^−^ and CD44^+^/CD24^+^ MDA-MB-231 subpopulations recapitulated the results obtained using SUM159 cells (Additional file 1: Fig. S5).

To determine whether NRP1 confers self-renewal potential to claudin-low cells, the effect of NRP1 inhibition on cell spheroid-forming capacity was investigated. Because even relatively low concentrations of siRNA transfection reagent were found to compromise spheroid-forming capacity, NRP1 inhibition was achieved using the anti-NRP1 monoclonal antibody, Vesencumab (Genentech/Roche), which targets the extracellular VEGF-binding b1/b2 domain of NRP1, versus IgG control (50 µg/ml each) [40]. Vesencumab treatment decreased the number of spheroids formed by SUM159 and Hs578T cell lines (Fig. 4l–m). Thus, these data suggest that high NRP1 expression is associated with a subpopulation of claudin-low tumors enriched for stem cell characteristics and promotes tumor cell self-renewal potential.

### NRP1 inhibition suppresses growth of SUM159 claudin-low orthotopic xenografts through an angiogenesis-independent mechanism

**Fig. 5:**
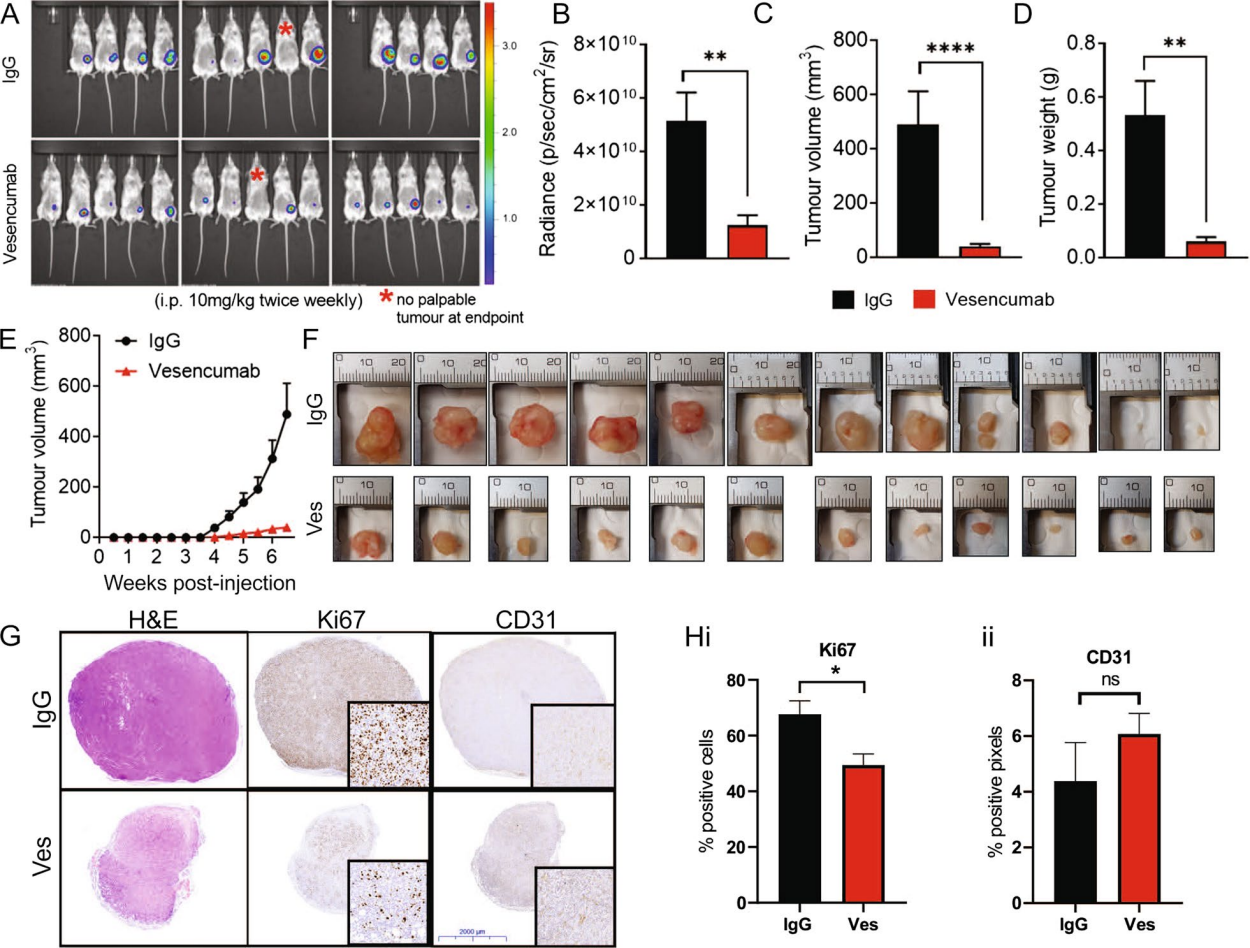
NRP1 inhibition suppresses in vivo growth of claudin-low SUM159 orthotopic xenografts. **a** Luciferase-derived luminescence signal from SUM159^luc^ primary tumors as imaged by the IVIS Spectrum In Vivo Imaging System at 7 weeks post-tumor inoculation. Endpoint (7 weeks) mean **b** tumor luciferase intensity, **c** tumor weight and **d** tumor volume. **e** Tumor growth curves in IgG control and Vesencumab groups. **f** Endpoint (7 weeks) tumors from IgG control and Vesencumab groups; two additional tumors from the Vesencumab-treated group were too small to collect. **g** Representative images of H&E staining and Ki67 and CD31 immunohistochemistry of Vesencumab and IgG control treated tumors, with quantification of **hi** Ki67 and **hii** CD31 staining across all tumors. ‘Ves’; Vesencumab. Error bars represent SEM; **p < 0.001; ****p < 0.0001. For **a**–**e**, *n* = 12–14. For **g**–**h**, *n* = 12

As NRP1 inhibition decreased spheroid formation, we speculated that NRP1 may enhance the in vivo tumor-initiating potential of claudin-low cells. Therefore, the efficacy of Vesencumab in suppressing in vivo SUM159 xenograft initiation and subsequent growth was investigated. Upon orthotopic injection of 1 × 10^6^ luciferase-expressing SUM159^Luc^ cells into the inguinal mammary fat pad, female NSG mice were treated twice weekly with Vesencumab or IgG (10 mg/kg intraperitoneal injection). At study endpoint 7 weeks after tumor inoculation, tumor-derived luciferase signal was greater in the IgG control versus Vesencumab group (Fig. 5a, b). Vesencumab treatment led to significant reduction in mean endpoint tumor volume and weight, as well as tumor growth compared to the IgG control group (Fig. 5c–f). Immunohistochemical staining of tumors collected at study endpoint revealed a significant reduction in the percentage of cells positive for Ki67 in the Vesencumab treated group (Fig. 5g and Hi). Although NRP1 is a known co-receptor for VEGF, no differences were observed in intra-tumoral vascularization between the treatment groups as measured by CD31 immunohistochemistry, suggesting that the tumor suppressive effects of Vesencumab were not primarily mediated by changes in tumor angiogenesis, but direct effects on tumor cells expressing NRP1 (Fig. 5g and Hii).

### NRP1 drives activation of the RAS-MAPK pathway in claudin-low cells

**Fig. 6:**
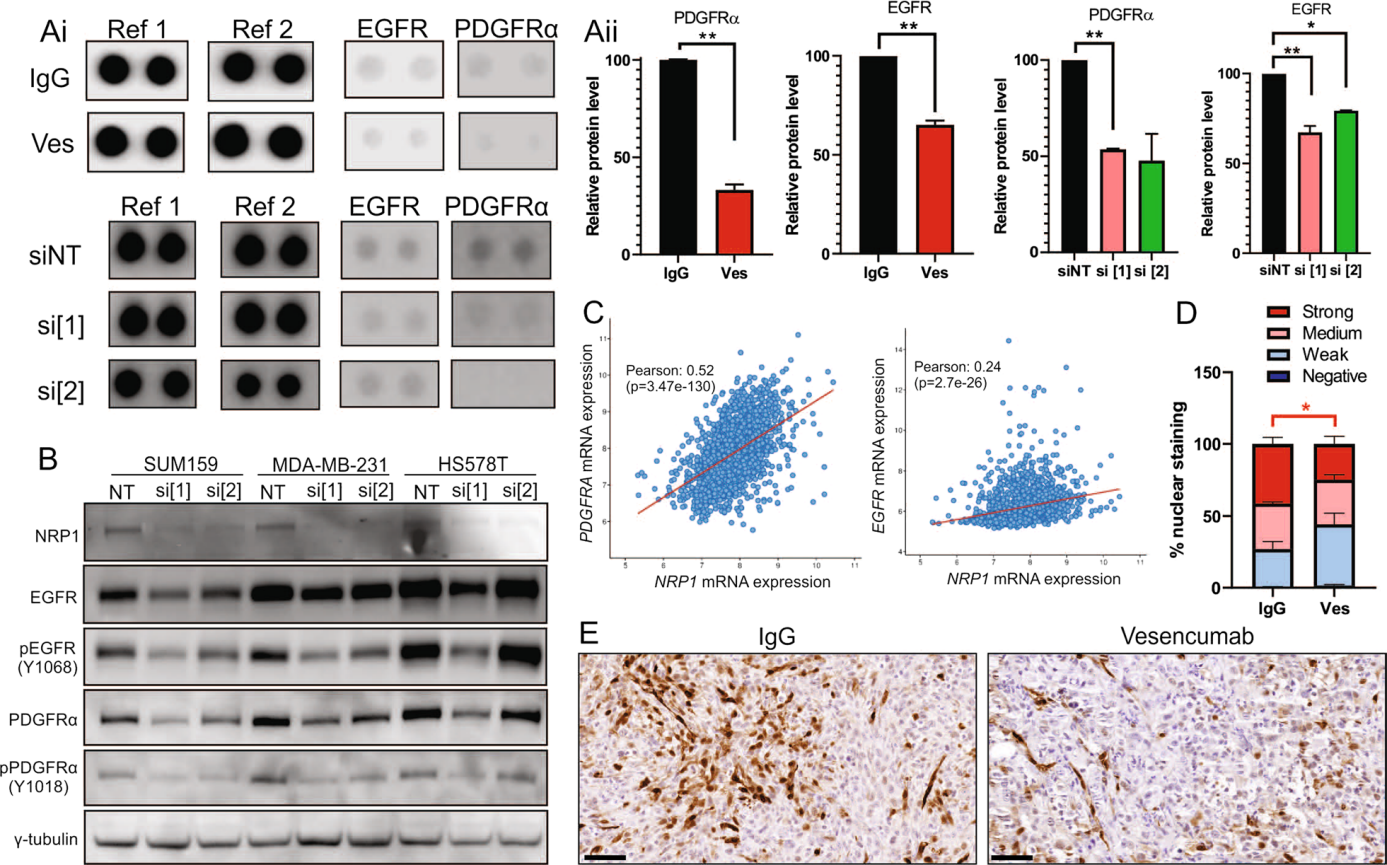
NRP1 inhibition suppresses EGFR and PDGFRα signaling in claudin-low cells. **ai** Receptor tyrosine kinase array showing EGFR and PDGFRα expression in SUM159 cells after 60 min treatment with 50 μg/mL IgG or Vesencumab (top panel), or 72 h after transfection with NRP1 targeting siRNA (siNRP1 [1] or siNRP1 [2]) versus non-targeting (NT) control (bottom panel), with **aii** corresponding densitometry. Ref1 and Ref2 represent positive controls. **b** Western blot showing NRP1, total EGFR, phospho-EGFR (pEGFR Y1068), total PDGFRα and phospho-PDGFRα (Y1018) expression in SUM159, MDA-MB-231 and HS578T cells after 72 h NRP1 knockdown versus NT control. **c** Correlation analysis between NRP1 and EGFR (right panel; Pearson: 0.24, *p* = 2.7e−26) and PDGFRα (left panel; Pearson: 0.52, *p* = 3.47e−130) mRNA expression in the METABRIC dataset (*n* = 1904) [25]. Data was obtained from cBioportal. **d** Immunohistochemical analysis of phosphorylated (T202/Y204) p42/44 levels in endpoint (7 weeks post-tumor inoculation) Vesencumab or IgG control treated tumors, with **e** representative images (right panel); scale bars = 50 µm, *n* = 5–6. Error bars represent SEM, *p ≤ 0.05; **p ≤ 0.01 versus control

Aberrant activation of RAS-MAPK signaling is a defining characteristic of claudin-low tumors, particularly the CL1 subtype [11]. Given the known role of NRP1 as a co-receptor for multiple receptor tyrosine kinases (RTKs), we sought to identify if NRP1 could mediate RTKs upstream of the RAS-MAPK pathway, such as EGFR or PDGFR. NRP1-dependent signaling pathways were interrogated using a phospho-RTK protein array, wherein Vesencumab treatment of SUM159 cells was indeed found to inhibit both EGFR and PDGFRα activation compared to control (Fig. 6ai, ii). These results were reproduced with NRP1 knockdown, where SUM159 NRP1 knockdown cells had reduced activation of EGFR and PDGFRα (Fig. 6ai, ii).

Phospho-antibody array findings were validated by Western blotting following both Vesencumab treatment (Additional file 1: Fig. S6) and NRP1 knockdown, wherein NRP1 knockdown was found to reduce both phosphorylated and total EGFR and PDGFRα (Fig. 6b; densitometry shown in Additional file 1: Fig. S6). The ratio of phospho/total receptor levels suggested that in most cases, NRP1 knockdown disproportionately decreased levels of phosphorylated EGFR/PDGFRα relative to total receptor expression (Additional file 1: Fig. S6), implying a greater effect on receptor activation rather than expression. However, additional mechanisms regulating expression of EGFR and PDGFRα are likely to be involved and will require further investigation.

Correlation analysis of clinical samples from the METABRIC dataset confirmed a significant positive association of *NRP1* mRNA expression with both *EGFR* and *PDGFRα* mRNA levels in patient tumors (Fig. 6c). Reduced levels of phosphorylated p42/44 MAPK were seen following NRP1 knockdown across most claudin-low cell lines and knockdown samples tested (Additional file 1: Fig. S6). Furthermore, levels of phosphorylated p42/44 MAPK were lower in endpoint tumors treated with Vesencumab than IgG control tumors, with a significantly lower percentage of nuclei exhibiting strong phospho-p42/44 staining by IHC (Fig. 6d, e). Pathologist assessment of phospho p42/44 staining suggested that differentially intense phospho p42/44-positive regions between treatment groups reflected predominantly tumor vascular walls, tumor endothelial-like cells (pseudo-vascular structures) and tumor cells.

Collectively, these data implicate NRP1 as an upstream regulator of RAS/MAPK signaling in claudin-low cells via activation of EGFR and PDGFRα.

## Discussion

Treatment of triple-negative breast cancer is currently limited by a lack of targeted therapies. A substantial proportion (25–39%) of triple-negative tumors classify as claudin-low. We report high NRP1 expression to be associated specifically with the claudin-low molecular subtype of breast cancer. This correlation was not due to immune or stromal cell infiltration in claudin-low tumors, as NRP1 expression correlated with a ‘core’ claudin-low signature enriched in EMT and cancer stem cell markers that characterize the claudin-low subtype [10].

NRP1 expression associated more strongly with the claudin-low up, rather than down, gene signature score, which is enriched in mesenchymal and stem cell markers. NRP1 expression was highest in CL1 subtype tumors, which most closely resemble mammary stem cells [11]. In the CL1 cell line Hs578T, NRP1 regulates expression of the EMT transcription factor ZEB1, which has been shown to be aberrantly over-expressed in the CL1 subtype, predisposes to malignant transformation in the absence of high levels of chromosomal instability and mediates mammary stem cell characteristics [11, 41, 42]. NRP1 was more highly expressed in a self-renewing CD44^+^/CD24^low^ population and conferred spheroid-initiating potential to claudin-low cells. Consistent with a known role in mediating integrin function in breast and other cell types, NRP1 was required to maintain expression of the mammary stem cell marker ITGA6 (CD49f) [43–45]. These data strongly suggest a role of NRP1 in normal mammary stem cell function, which is dysregulated following neoplastic transformation, supported by the finding that NRP1 depletion induces defects in mammary epithelial cell development [46].

The NRP1-targeted antibody Vesencumab was able to potently inhibit the growth of SUM159 orthotopic xenografts, whereby the mean volume of Vesencumab-treated tumors at the 7-week study endpoint was 12.8-fold smaller than IgG treated tumors. The main effects of Vesencumab were likely due to direct anti-tumor effects on tumor cells and largely independent of changes to angiogenesis, as no difference in CD31-positive intratumoral vasculature was observed between Vesencumab and IgG treated tumors. In our model, Vesencumab was administered at the time of tumor xenografting until study endpoint to establish the effects of NRP1 inhibition on tumor latency and growth kinetics, as suggested by its association with a mammary stem cell population. In future experiments, it will be important to determine the tumor-suppressive potential of Vesencumab when administered to animals with established tumors or after tumor resection, to more closely resemble the timing of therapeutic intervention in future clinical trial testing.

As all the claudin-low cell lines used in this study were basal-like, further studies assessing the efficacy of NRP1 inhibition across claudin-low models of different intrinsic subtypes will also be of interest. As high NRP1 expression was particularly associated with poor prognosis in ER negative tumors, we suggest that the greatest clinical utility of NRP1 inhibition is likely to be in patients with claudin-low TNBC.

Evidence is accumulating that aberrant activation of the RAS/MAPK pathway is a key mechanism capable of driving transition to a claudin-low phenotype [11, 47]. Despite demonstrably high RAS pathway signaling in a substantial proportion of breast tumors, RAS mutations occur relatively infrequently in human breast cancers, suggesting that engagement of the RAS pathway may be driven by alternate cooperative mechanisms [48]. Here, we show that NRP1 drives MAPK signaling in claudin-low cells via activation of upstream receptor tyrosine kinases. Changes in phospho p42/44 staining in Vesencumab-treated tumors suggested that NRP1 may regulate the RAS/MAPK axis in tumor vascular walls and endothelial-like cells. However, future experiments to validate NRP1 regulation of endothelial cell function and vascular mimicry in claudin-low tumors via the RAS/MAPK axis will be required to confirm or disprove these speculations.

NRP1 has recently been implicated in resistance to cancer therapies including oncogene-targeted therapies and chemotherapy through activation of bypass survival pathways, including receptor-tyrosine kinase pathways such as HER2, EGFR and IGF1R [18, 21, 43]. Thus, blocking NRP1-mediated activation of a spectrum of therapy-induced bypass survival pathways beyond just RAS/MAPK, either as a single agent or alongside standard of care chemotherapy regimens, may provide superior control over adaptive resistance mechanisms and improve the durability of therapy responses.

## Conclusions

There is a shortage of durable targeted therapies available to treat TNBC, which lack expression of the clinically actionable estrogen/progesterone receptors and HER2 amplification. Our findings identify NRP1 over-expression as a feature of claudin-low breast cancers, a substantial proportion of TNBC. Taken together, these data strongly implicate NRP1 in claudin-low tumor progression and provide the preclinical rationale for future studies assessing NRP1 inhibition as a novel therapeutic strategy for this aggressive BrCa subtype.

## Supplementary Information

**Additional file 1.** Supplementary figures 1–6.

## Data Availability

All data generated or analyzed during this study are included in this published article and its Additional file 1.

## Abbreviations

BrCa: Breast cancer
EGFR: Epidermal Growth Factor Receptor
ER: Estrogen Receptor
CL: Claudin-low
CoreCL: Core claudin-low
CSC: Cancer stem cells
DMFS: Distant metastasis-free survival
EMT: Epithelial-mesenchymal transition
GSE: Gene set enrichment
GSVA: Gene set variation analysis
HER2: Human Epidermal Growth Factor Receptor 2
IHC: Immunohistochemistry
ITGA6: Integrin alpha-6 (CD49f)
KM: Kaplan–Meier
Luc: Luciferase
LumA: Luminal A
LumB: Luminal B
METABRIC: Molecular Taxonomy of Breast Cancer International Consortium
NRP1: Neuropilin 1
NSG: NOD scid gamma
NT: Non-targeting
PAM50: Predication Analysis of Microarray 50
PDGFR: Platelet-derived growth factor receptor
PR: Progesterone Receptor
RAS-MAPK: Rat Sarcoma Virus-Mitogen Activated Protein Kinase
RFS: Relapse-free survival
TCGA: The Cancer Genome Atlas
TNBC: Triple-negative breast cancer
ZEB1: Zinc finger E-box-binding homeobox 1
