# Supplementary material for: Neuropilin-1 is over-expressed in claudin-low breast cancer and promotes tumor progression through acquisition of stem cell characteristics and RAS/MAPK pathway activation
Source: Breast Cancer Res. 2022 Jan 25;24:8. doi: 10.1186/s13058-022-01501-7 (PMC8787892; doi:10.1186/s13058-022-01501-7)
Supplement: Supplementary file 1 — Additional file 1. Supplementary figures 1–6. [file 13058_2022_1501_MOESM1_ESM.docx]

**Supplementary Materials**


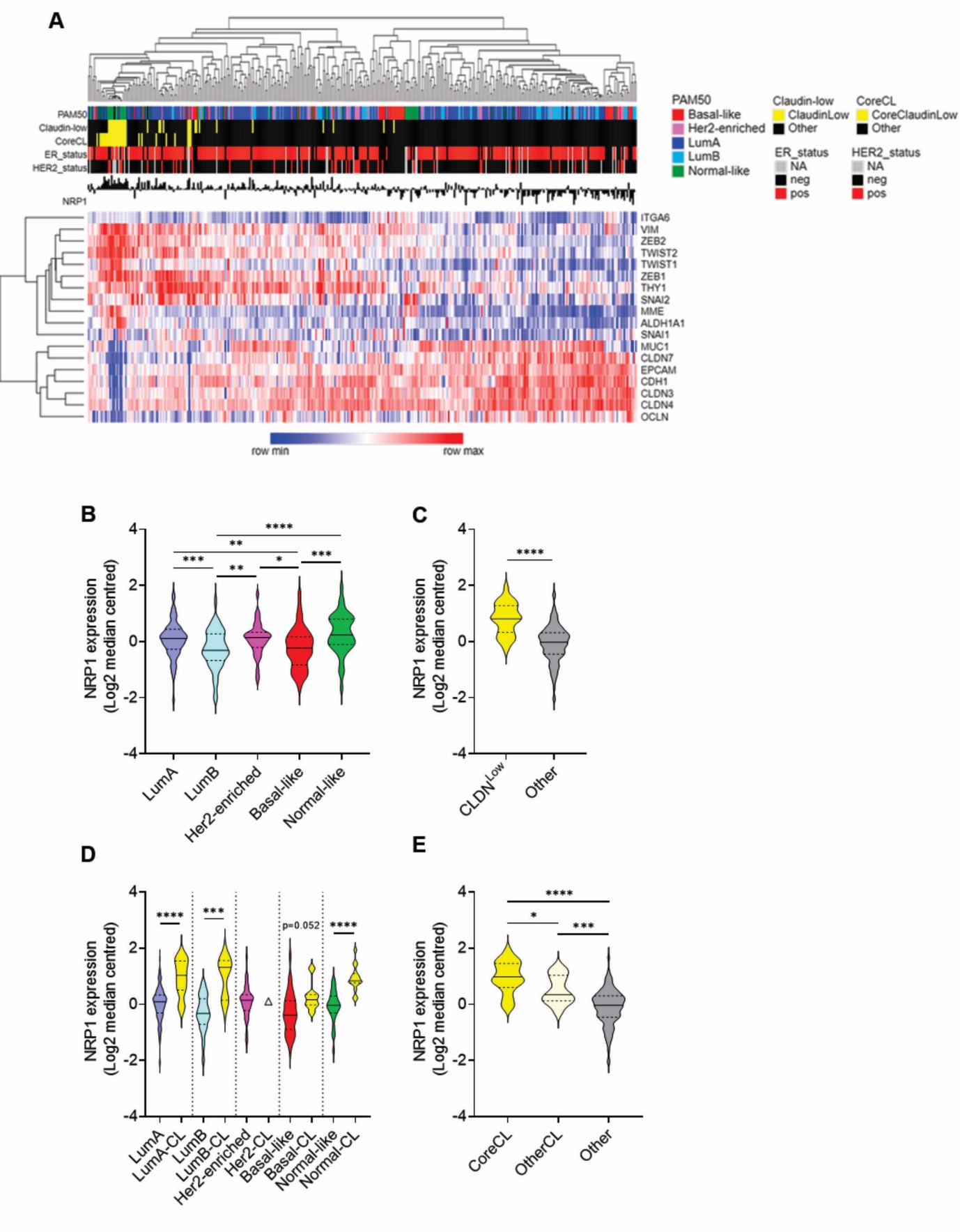


***Figure S1. Association of NRP1 mRNA expression with breast cancer subtypes in the Oslo2 patient dataset.***

***(A)*** *Heatmap showing NRP1 expression association with PAM50, claudin-low, core claudin-low (CoreCL), ER and HER2 tumor status, as well as core claudin-low signature genes in the Oslo2 cohort (425 patients) (34). Association of NRP1 mRNA expression with* ***(B)*** *intrinsic breast cancer subtypes and* ***(C)*** *claudin-low (CLDNl^ow^) samples in the Oslo2 patient dataset.* ***(D)*** *NRP1 mRNA expression across intrinsic subtypes subdivided into claudin-low (CL) and non-claudin-low tumors.* ***(E)*** *NRP1 mRNA expression in core claudin-low (CoreCL), non-core claudin-low (OtherCL) and non-claudin-low tumors (10). Error bars represent SEM, * P ≤0.05; ** P ≤ 0.01; *** P ≤ 0.001; **** P ≤ 0.0001.*

***Figure S2****
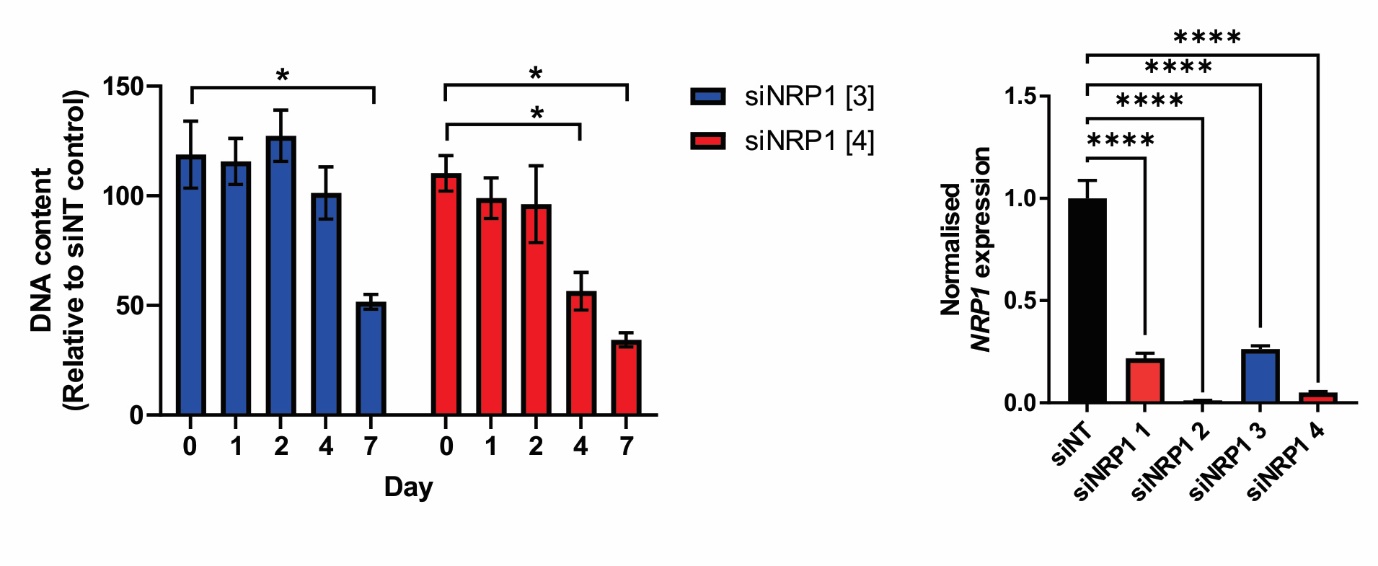
****: Inhibition of MDA-MB-231 cell proliferation by two additional NRP1-targeted siRNA sequences.*** *Cell viability of MDA-MB-231 cells at 0, 1, 2, 4 and 7 days after transfection with NRP1 siRNA [3] (5’ - 3’ cttcatctattcccaagct) or [4] (5’ – 3’ ggtttctcagcaaactaca)) relative to siNT as measured by CyQuant™ DNA quantification assay (left panel), and qPCR showing NRP1 expression 72 hours post siRNA transfection in siNRP1 and control cells (right panel)****.*** *N=3, error bars represent SEM, * P ≤0.01; **** P ≤ 0.0001.*

***
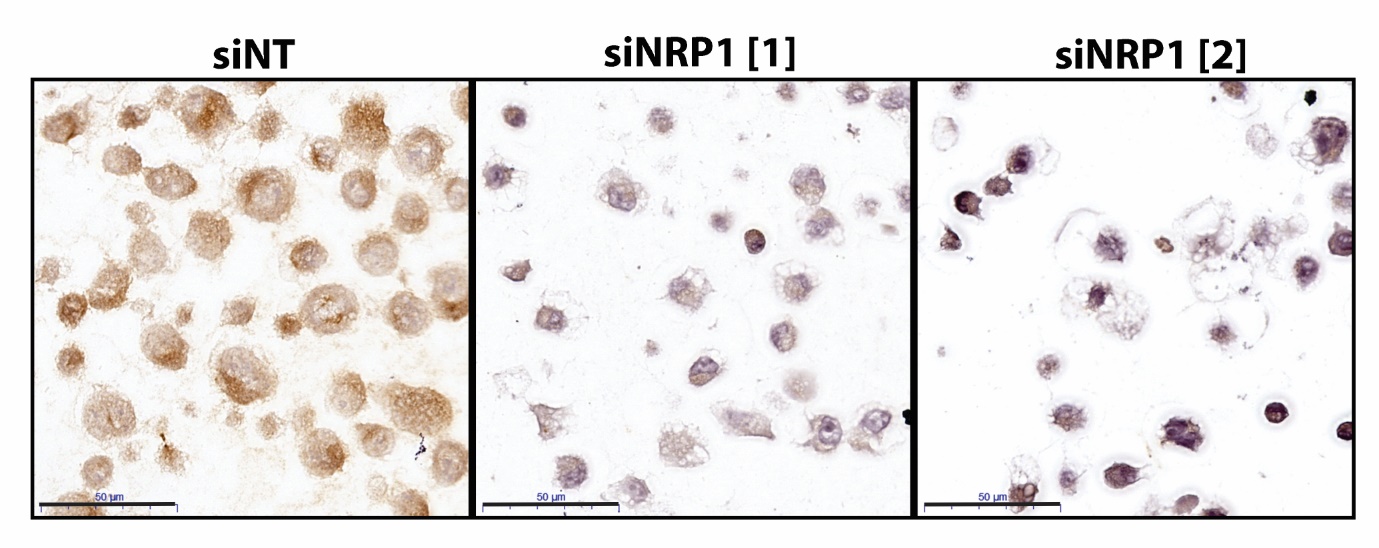
Figure S3: Immunohistochemical validation of NRP1 antibody specificity*.** *Immunohistochemistry of SUM159 cells 72 hours after transfection with NRP1-targeted siRNA (siNRP1 [1] and siNRP1 [2]) or non-targeting control (siNT). Antibody (Sigma Aldrich, HPA030278) was used at 4 µg/ml. Scale bars = 50 µM.*

*
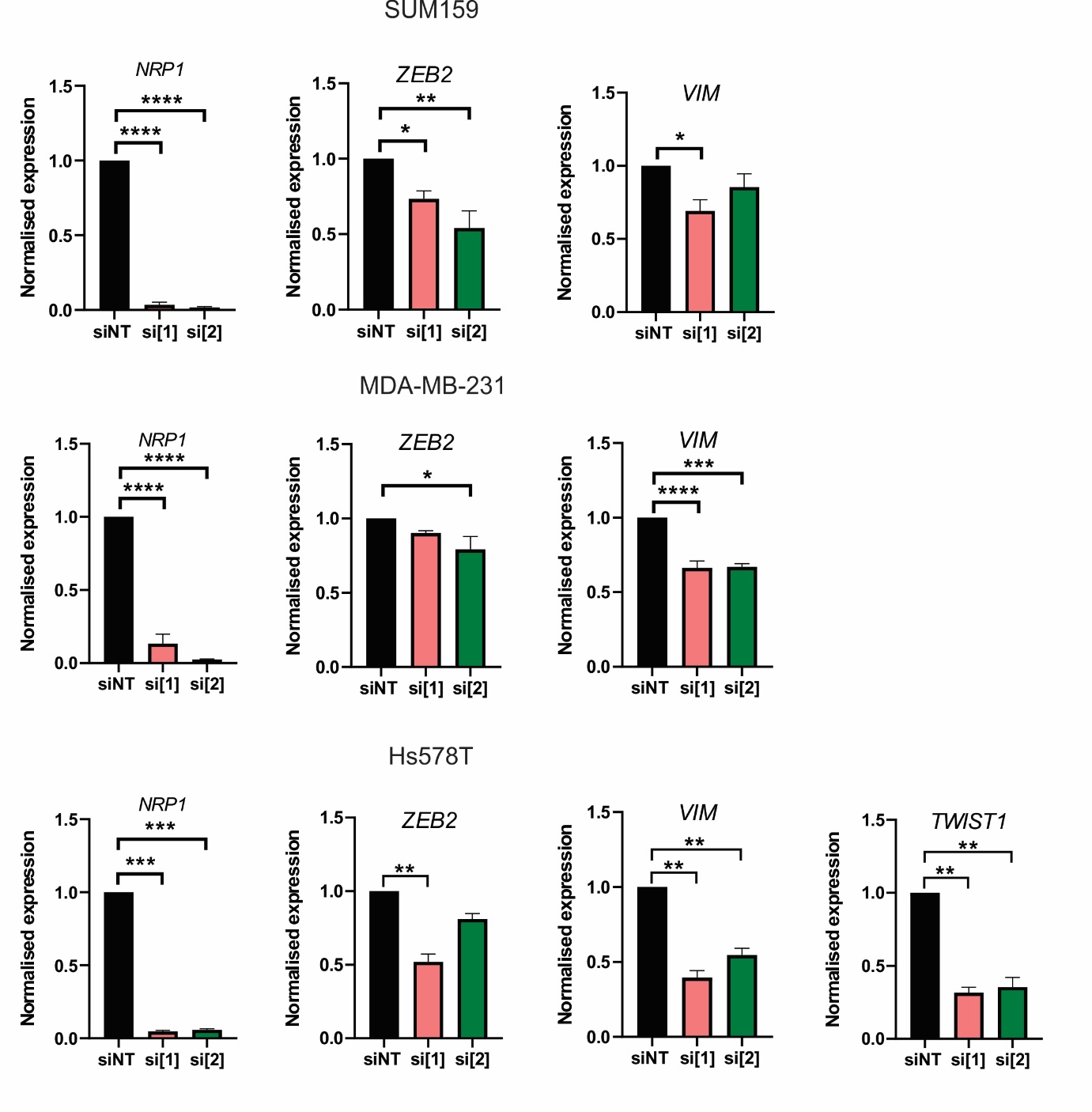
****Figure S4: Expression of EMT markers following NRP1 siRNA knockdown in claudin-low cells****. Expression of NRP1, ZEB2, VIM and TWIST1 (Hs578T cells only) transcript levels in SUM159 (top panel), MDA-MB-231 (middle panel) and Hs578T (bottom panel) cells 72 hours post-transfection with non-targeting (NT) control or NRP1-targeting (si[1] and si[2]) siRNA sequences. N=3, error bars represent SEM, * P ≤0.01; ** P ≤ 0.001, *** P ≤ 0.0001 and **** P ≤ 0.00001 versus control.*

**
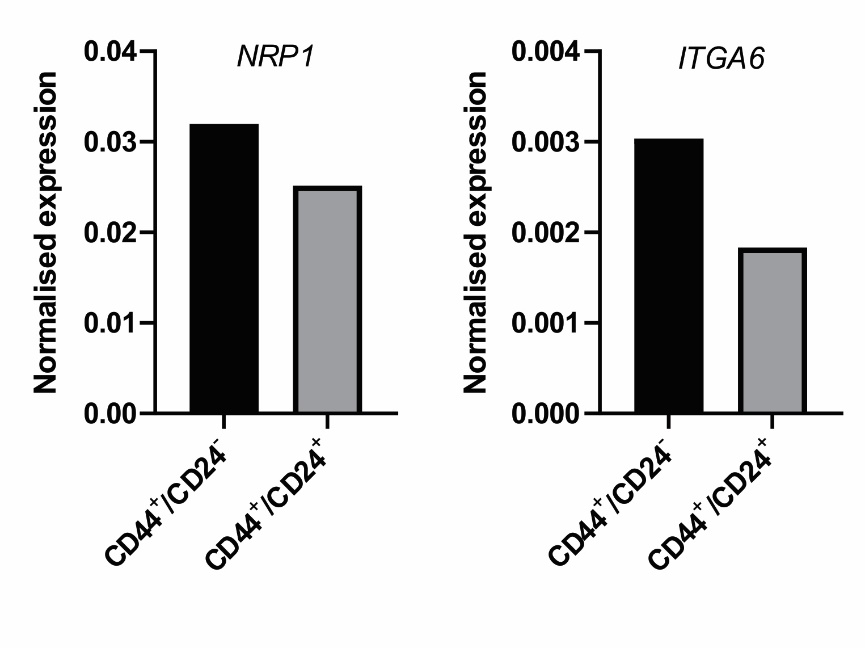
**

***Figure S5: NRP1 mRNA expression in CD24^+/-^ MDA-MB-231 cell populations.*** *qPCR analysis showing expression of NRP1 and ITGA6 mRNA in FACS-sorted CD44^+^/CD24^lo^ and CD44+/CD24^hi^ populations of MDA-MB-231 cells (n=1).*

**
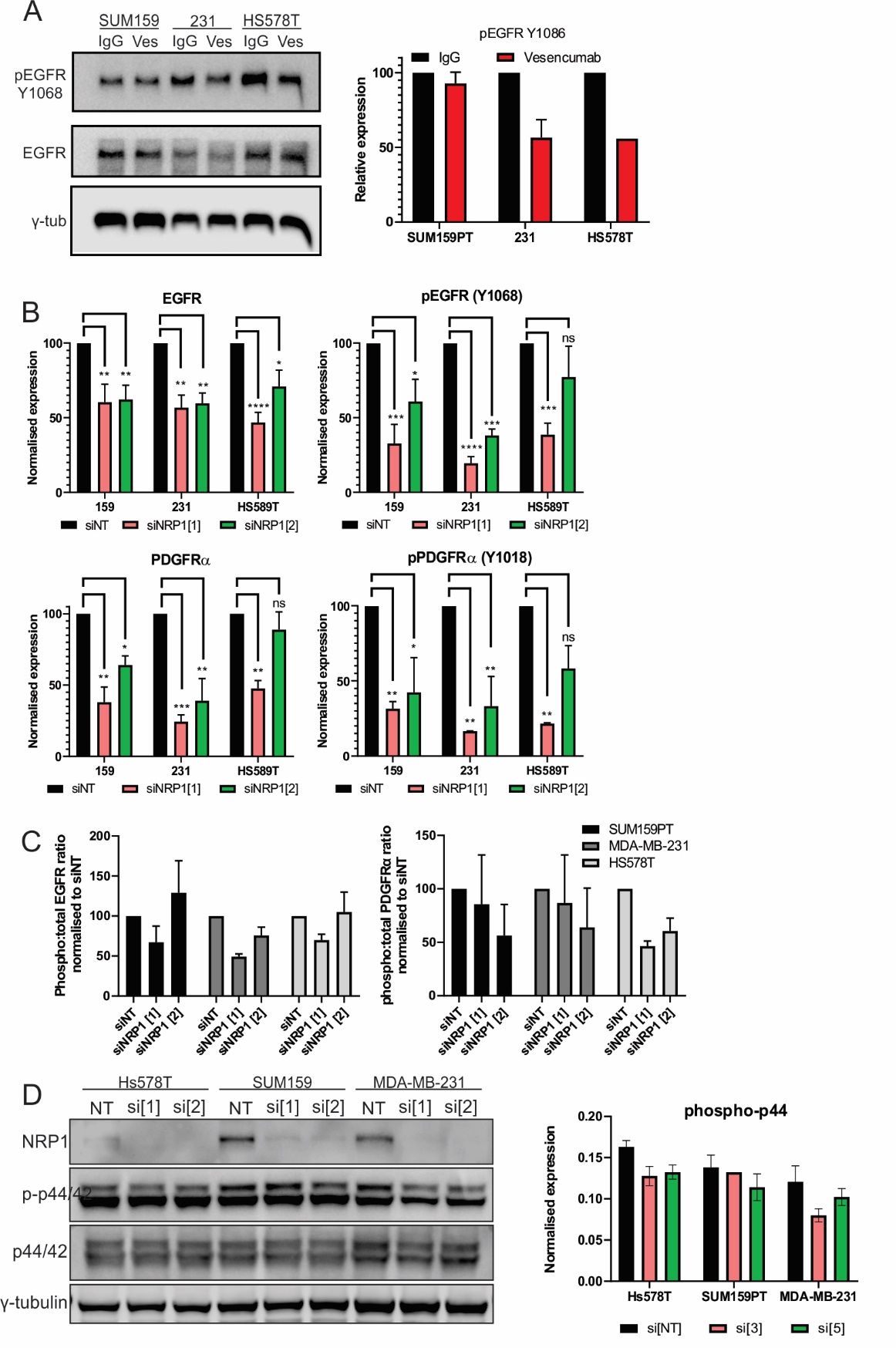
**

***Supplementary Figure S6: A)*** *Western blotting and densitometry of phospho-EGFR (Y1086) levels in SUM159, MDA-MB-231 and Hs578T cells following 60-minute treatment with 50 µg/ml Vesencumab or IgG control.* ***B)*** *Densitometry for EGFR, phospho-EGFR (Y1086), PDGFRα, phospho-PDGFRα (Y1018) and phospho-p42/44 (T202/Y204) western blotting in Figure 6 (n=2-4).* ***C)*** *Densitometry of western blot data showing ratio of phospho : total EGFR (left panel) and PDGFRα (right panel) protein levels in NRP1 knockdown (siNRP1) SUM159, MDA-MB-231 and Hs578T cells relative to non-targeting (siNT) control.* ***D)*** *Western blot and densitometry showing expression of phosphorylated (T202/Y204)* *and total p42/44 MAPK in expression in HS578T, SUM159 and MDA-MB-231 cells after 72 hours NRP1 knockdown versus NT control. Densitometry was normalized to housekeeping control (GAPDH or γ-tubulin).*
